# Analyzing gene-based apoptotic biomarkers in insomnia using bioinformatics

**DOI:** 10.1097/MD.0000000000040965

**Published:** 2025-01-17

**Authors:** Wenwen Zhu, Xingchun Yang, Nanxi Li, Bin Zhang, Lishan Huang, Hanxing Cheng, Xiao Wu, Dechou Zhang, Sen Li, Houping Xu

**Affiliations:** a Geriatric Department, The Affiliated Traditional Chinese Medicine Hospital, Southwest Medical University, Luzhou, Sichuan, China; b Department of Acupuncture and Tuina, Affiliated Hospital of Traditional Chinese Medicine, Southwest Medical University, Luzhou, Sichuan, China; c Department of Orthopedic Surgery, Division of Spine Surgery, Nanjing Drum Tower Hospital, Affiliated Hospital of Medical School, Nanjing University, Nanjing, Jiangsu, China.

**Keywords:** apoptosis, biomarkers, insomnia

## Abstract

Insomnia is increasingly common and poses significant health risks. The aims of this study are to identify apoptosis-related genes and potential biomarkers for insomnia and to find new therapeutic targets. Insomnia gene expression profiles were downloaded from the Gene Expression Omnibus database, and differentially expressed genes in normal and insomnia samples were identified by limma rapid differential analysis, and then the major modular genes with clinical relevance to insomnia were analyzed using the Weighted Gene Co-Expression Network Analysis, and intersections were obtained with the differentially expressed genes as well as with apoptotic gene databases. We validated apoptosis-related differentially expressed genes, enriched and analyzed the specific biological process of insomnia and related signaling pathways. In addition, we constructed a protein–protein interaction network and obtained Top10 hub genes using Cytoscape. We selected 3 of them as hub genes and compared their expression in normal hippocampal neuronal cells and hippocampal neuronal cells of the model group exposed to corticosterone induction by Western Blot and qRT-PCR experiments. A total of 190 differentially expressed apoptosis-related genes were identified in insomnia, and BCL2, SOCS3, and IL7R were identified as important hub genes. Enrichment analysis showed that the occurrence of apoptosis in insomnia was mainly related to “PI3K-Akt signaling pathway,” “JAK-STAT signaling pathway,” “P53 signaling pathway” and so on. GO analysis showed that apoptosis in insomnia was mainly related to “immune response,” “T cell differentiation in thymus,” and “positive regulation of MAPK cascade.” Western Blot and qRT-PCR experiments showed that BCL2, SOCS3, IL7R antiapoptotic indexes were under-expressed in modeled hippocampal neuronal cells compared to normal hippocampal neuronal cells. This study emphasizes the role of apoptosis-related genes in insomnia and preliminarily predicts that the occurrence of insomnia is closely related to apoptosis. Compared to the normal group, the antiapoptotic ability of hippocampal neurons in the model group is reduced. Although BCL2 has been studied in the context of sleep deprivation, SOCS3 and IL7R have not yet been explored in insomnia. Insomnia and sleep deprivation involve similar pathways, but due to different mechanisms and types of insomnia, gene expression may vary.

## 1. Introduction

Insomnia is a sleep disorder characterized by difficulty falling asleep, early waking, excessive dreaming, and daytime fatigue. According to studies, over 27% of people worldwide will experience sleep disorders in 2021, and 29.8% of Americans will have trouble falling asleep between 2017 and 2022.^[1]^ Insomnia has grown to be a significant public health issue that is widely monitored worldwide. Chronic insomnia can cause serious damage to health, disrupting immune function, reducing cognitive function, and greatly increasing the risk of other diseases and financial burden. Insomnia is commonly categorized into primary insomnia and co-morbid insomnia secondary to other diseases, acute insomnia and chronic insomnia according to the time of onset, and childhood insomnia, adolescent insomnia, peri-menopausal women’s insomnia, and seniors’ insomnia according to the population. In general, women experience insomnia more frequently than men, and older adults experience insomnia more frequently than younger age groups.^[2,3]^ Although the precise cause of insomnia, a collection of closely related neuro-psychological diseases, is unknown, certain risk factors for insomnia have been found. There are correlations with age and gender, and factors including behavioral, genetic, psychological, and environmental factors are implicated. Insomnia has been linked to metabolic disruptions and elevated electroencephalogram activity during Non-Rapid Eye Movement sleep.^[4,5]^ These include elevated systemic metabolic rate during sleep and wakefulness, elevated cortisol and adrenocorticotropic hormone early in sleep, and reduced levels of pentraxytryptophan and gamma aminobutyric acid during insomnia. However, the diagnosis of insomnia is still largely based on patients’ subjective accounts, and decisive diagnostic evidence is still lacking. Currently, cognitive behavioral therapy is advised as the first-line treatment for insomnia.^[6]^ Nevertheless, patient compliance is low, and the applicability is highly limited due to the lack of specialized physicians, high time, and financial constraints. The most often used clinical pharmacological treatments, including diazepam and eszopiclone, are benzodiazepines that are controversial because of their serious side effects, which include addiction and withdrawal symptoms. There are other therapies as well, such acupuncture, Chinese medicines, melatonin, etc, but the evidence for these methods is not strong enough. In general, tailored treatments with fewer side effects are lacking, as are biomarkers that can be used as a basis for diagnosis of insomnia. Current research is desperately needed to provide a more effective, safer, and focused treatment.

Recent studies have uncovered a complicated relationship between apoptosis and insomnia, suggesting a possible molecular connection between the 2.^[7]^ Significantly greater amounts of apoptosis markers are seen in insomnia sufferers, indicating that insomnia may influence the body’s health by encouraging apoptosis.^[8]^ An elevated inflammatory response, which is believed to be a significant inducer of apoptosis, frequently coexists with insomnia.^[9]^ Patients with insomnia have higher levels of inflammatory mediators that can cause apoptosis, such as interleukin (IL) 6 and tumor necrosis factor-α. Increased oxidative stress, which has also been connected to the onset of apoptosis, can also result from insomnia.^[10,11]^ These apoptotic processes may impact particular cell types, particularly neurons, rather than just cells in general. Long-term sleep deprivation damages neurons by raising the expression of proteins linked to apoptosis in the brain.^[12]^ Additional research has demonstrated that sleeplessness may influence particular apoptotic signaling pathways, including the death receptor and mitochondrial pathways, which may result in neuronal apoptosis. For instance, BCL2 family proteins, such as BAX and BCL2, are essential for controlling apoptosis and cell survival.^[13–16]^ Thus, a thorough investigation of the connection between apoptosis and insomnia not only aids in understanding the basic mechanisms underlying the condition, but it may also yield novel approaches to its intervention and treatment. The detrimental consequences of sleeplessness can be lessened and neuroprotection can be encouraged by modifying apoptotic pathways.^[17]^ Therefore, our study aims to identify key apoptotic targets in insomnia, which may help to identify new therapeutic targets.

The hippocampus is a subcortical structure that has been implicated in the pathogenesis of insomnia and plays a key role in memory formation, navigation and cognition.^[18]^ Studies have shown that prolonged exposure to stress, wakefulness and insomnia can lead to impaired hippocampal structure and function, including memory formation, long-term potential and neurogenesis.^[19]^ Bilateral hippocampal volume reduction was found in patients with insomnia, suggesting that hippocampal atrophy is negatively correlated with sleep quality.^[20]^ Prolonged sleep deprivation may exacerbate hippocampal neurobehavioral function by promoting apoptosis, which inhibits the growth and survival of hippocampal neuronal cells and disrupts neurogenesis.^[21]^

The ability to detect the expression of thousands of genes has been made possible by recent rapid improvements in gene microarray technology and bioinformatics analysis. This has allowed us to better understand the pathophysiological role of genes in disease. Recently, high-throughput sequencing technologies have unveiled fresh insights into the development of disease and the identification of biomarkers.^[22]^ Based on bioinformatic analyses, we evaluated the signaling pathways of insomnia diseases by examining the GSE82114 dataset to determine the expression profiles of apoptosis-related genes between normal subjects and insomnia patients, and assessed insomnia diseases based on apoptosis-related genes. In addition, we conducted Western blot and PCR experiments to validate the expression of apoptosis-related genes. This validation aims to provide valuable scientific information to aid in the development of insomnia treatments and the identification of potential therapeutic targets.

## 2. Materials and methods

### 2.1. Microarray data sources and differential gene analysis

Gene expression microarrays were obtained from the Gene Expression Omnibus (GEO) database (https://www.ncbi.nlm.nih.gov/geo/). Chip data acquisition platform is GPL15331, access number is GSE82114 (INSOMNIA = 34, control = 15), all with human sources.^[23]^ The microarray matrix data was converted to gene name matrix expression data using the Gene Name Conversion Tool in Sangerbox (version 3.0, http://sangerbox.com). The “limma” tool (version 3.40.6) was then selected for differential gene analysis to identify differential genes between the insomnia samples and the normal control samples. Specifically, we obtained the expression profiling dataset by firstly performing log2 transformation on the data, multiple linear regression using lmFit function, further using eBays function for compute moderated t-statistics, moderated F-statistic, and log-odds of differential expression by empirical Bayes moderation of the standard errors towards a common value, and finally obtain the differential significance of each gene. The thresholds for differentially expressed genes were set at a multiplicity of difference of 1.5-fold and *P*-value < .05,^[24,25]^ with positive numbers indicating up-regulation of differentially expressed genes (DEGs). Similarly, a multiplicity of difference of 1.5-fold and a *P*-value < value of .05, and a negative number indicates down-regulation of differentially expressed genes. Volcano and heat maps were utilized to show the results of differentially expressed genes.^[26]^

### 2.2. Weighted gene co-expression network analysis

The systems biology strategy Weighted Gene Co-Expression Network Analysis (WGCNA) was used to explore the correlation between genes.^[27]^ First, the median absolute deviation of each gene was determined by removing 50% of the gene with the smallest median absolute deviation. Second, the DEG expression matrix was filtered by utilizing the good samplegenes function to eliminate the unqualified genes and samples, and the scale-tree co-expression network was established. The soft threshold power (β) was selected based on the scale-free topology criterion (threshold set at *R*² > 0.9) to compute neighbor-joining relationships from the co-expression similarity. Neighborhoods were then transformed into topological overlap matrices to determine their gene proportions and differences. Next, the modules were detected using hierarchical clustering and dynamic tree cut function. The average chain hierarchical clustering method was used to divide the genes expressing the same spectrum into multiple gene modules. Finally, the dissimilarity of the genes characterized by the modules was calculated, the cut line of the module tree was selected, and multiple modules were combined together for further study. Millions of genes are entered into modules according to their expression patterns, and each module has genes with common expression patterns. Use “VennDiagram” (version 1.6.20) to find overlaps between core module genes and DEGs.

### 2.3. Insomnia disease genes and apoptosis gene acquisition

In order to study the targets and effects of insomnia disease, GeneCards (https://www.genecards.org/), Comparative Toxicogenomics Database (https://ctdbase.org/) disease database was searched with the “Insomnia” as the keyword, a search was performed to obtain the target proteins for the action of the disease.^[28]^ Genes related to apoptosis were obtained from the literature and GeneCards database.^[29,30]^ The intersection of GeneCards, differential gene sets, and WGCNA to obtain genes related to apoptosis in insomnia diseases was taken, and the intersecting genes were initially identified as important target genes for the development of insomnia.

### 2.4. Enrichment analysis

Import the hub gene set into Sangerbox (version 3.0, http://sangerbox.com) BioCloud, select Enrichment Analysis in the Tool Center, and limit the species to “H. sapiens.” Enter the Gene Symbol of the hub gene gene set in the common parameters and submit. Finally, we obtained the GO enrichment analysis and KEGG database pathway analysis of the hub genes and presented the results in different graphs.^[31,32]^

### 2.5. Protein–protein interaction network analysis

Protein–protein interactions (PPIs) underlie most biological processes in living cells and are essential for understanding cellular physiology in normal and disease states. In this study, PPI network analysis was performed using the string database (http://string-db.org/) on the set of intersecting genes obtained for the species restricted to “Homo sapiens” with a confidence value > 0.4. The PPI network was constructed by Cytoscape software (version 3.9.1).^[33]^ In addition, the CytoHubba algorithm, a plug-in for Cytoscape software, was used (Density of Maximum Neighborhood Component, Maximum Neighborhood Component, Edge Percolated Component, Closeness, Betweenness, ClusteringCoefficient, EcCentricity, Radiality, Stress, BottleNeck) to obtain the key gene set.^[34]^ To further pinpoint the genes acting during insomnia, the key gene set was screened using the CytoHubba algorithm to obtain 10 key genes, and finally BCL2, SOCS3, and IL7R were obtained as pivotal genes by Degree grading.

### 2.6. Validation of key genes and pathways

We subjected potential hub target genes to Gene Set Enrichment Analysis (GSEA) to elucidate the key signaling pathways involved in the regulation of these genes.^[35]^ We obtained the GSEA software (version 3.0) from the GSEA (DOI: 10.1073/pnas. 0506580102, http://software.broadinstitute.org/gsea/index.jsp) website and based on the expression level of statistically significant pivotal genes, we divided the samples were divided into high (≥50%) and low (<50%) expression groups. And downloaded c2.cp.kegg.v7.4. symbols.gmt subset from the Molecular Signatures Database (DOI:10.1093/bioinformatics/btr260, http://www.gsea-msigdb.org/gsea/downloads.jsp) to assess relevant pathways and molecular mechanisms. Based on gene expression profiles and phenotypic grouping, the minimum gene set was set to be 5, the maximum gene set to be 5000, and 1000 times resampling, *P* value of <.05 (as needed) and a FDR of <0.25 (as needed) were considered statistically significant.

### 2.7. Western Blot, qRT-PCR validation of hub genes

#### 2.7.1. Material preparation

Hippocampal neuronal cells were purchased from Procell, Canton, MA (CL-0697). Corticosterone was purchased from Supelco, Bellefonte, PA (C-117). Cell Counting Kit-8 (CCK8) assay kit was purchased from DOJINDO, Rockville, MD (VH597). GAPDH (bsm-52262R), BCL2 (bs-0032R), IL7R (bs-24840R), SOCS3 (bs-20752R) primary antibodies were purchased from Bioss, Woburn, MA. quantitative PCR (qPCR) kit was purchased from Vazyme, Nanjing, China (Q711-02).

#### 2.7.2. CCK8 viability assay

We used Dulbecco’s Modified Eagle Medium supplemented with 10% fetal bovine serum and 1% penicillin–streptomycin mixed solution. This composition ensures optimal growth and survival of HT22 cells. Hippocampal neuronal cells were cultured to 80% density, and the cells were inoculated in 96-well plates with 3000 cells per well and cultured at 37 °C cytosolic attachment for 24 hours in a 5% CO_2_ incubator. After intervention in 200 μL of medium containing different concentrations of corticosterone (0 mM, 50 mM, 100 mM, 200 mM, 300 mM), cells were cultured for 24 hours.^[36]^ Cell proliferation function was determined by adding 10 μL of CCK-8 solution to each well. Ten microliters of CCK-8 solution was added to each well and incubated for 2 hours in the incubator. The OD value at 450 nm was read using a multifunctional enzyme marker. The best intervention concentration was selected as the most modeling group for subsequent experiments.

#### 2.7.3. Western Blot, qRT-PCR validation

Protein and RNA differences between the model and control groups were detected by Western Blot, qRT-PCR. The approximate steps of Western Blot (WB) were as follows: Hippocampal neuronal cells from each group were rinsed twice with cold phosphate-buffered saline and then lysed uniformly with 1 mL of ice lysis buffer. The supernatant was collected and incubated on ice for 20 minutes before being centrifuged at 12,000 rpm for 20 minutes at 4 °C. Total protein content was determined using a bicinchoninic acid protein assay kit. The same amount of protein was extracted from each sample using SDS-PAGE; the isolated proteins were then transferred to a polyvinylidene difluoride membrane. After sealing in 5% skim milk for 2 hours at room temperature, the membrane was incubated with primary antibody at 4 °C overnight. The polyvinylidene difluoride membrane was then treated with secondary antibody for 2 hours at room temperature. GAPDH was selected as the reference protein. Images were densitometrically analyzed using ImageJ software version 1.8.

*qRT-PCR steps*: Total RNA was extracted from cells using the Total RNA Extraction Kit. One gram of total RNA was then reverse transcribed into cDNA using the iScript cDNA Synthesis Kit. Relative mRNA levels were calculated, GAPDH normalized using the 2‐ΔΔCt technique and analyzed by real-time fluorescence qPCR using the Bio-RadCFX96 device for real-time fluorescence qPCR analysis.

*PCR conditions*: initial denaturation: 95 °C for 5 minutes. Cycling conditions: 40 cycles including denaturation at 95 °C for 30 seconds, annealing at 55 to 60 °C for 30 seconds, and extension at 72 °C for 30 seconds. Final extension: 72 °C for 10 minutes.

*cDNA synthesis*: reverse transcription conditions: 42 °C for 60 minutes, followed by inactivation at 70 °C for 5 minutes. Reaction system: volume of PCR reaction mixture: total volume of 20 μL, consisting of 10 μL 2 × PCR master mix, 1 μL primers, 1 μL cDNA, with the remaining volume made up with RNase-free water.

We obtained the specific information about the primers from Sangon Biotech. The final primer sequence list of genes encoding BCL2, SOCS3, IL7R was obtained. The primer sequence numbers are listed below:

GAPDH-F 5′ AGGTCGGTGTGAACGGATTTG 3′

GAPDH-R 5′ TGTAGACCATGTAGTTGAGGTCA 3′

BCL2-F 5′ GTCGCTACCGTCGTGACTTC 3′

BCL2-R 5′ CAGACATGCACCTACCCAGC 3′

SOCS3-F 5′ ATGGTCACCCACAGCAAGTTT 3′

SOCS3-R 5′ TCCAGTAGAATCCGCTCTCCT 3′

IL7R-F 5′ GCGGACGATCACTCCTTCTG 3′

IL7R-R 5′ AGCCCCACATATTTGAAATTCCA 3′

### 2.8. Statistical analysis

IBM SPSS Statistics 26 and R software (Version 2.15.3) was used for statistical analysis. Normally distributed data were analyzed using Student test and non-normally distributed data were analyzed using Mann–Whitney *U* test. * *P* < .05, ** *P* < .01, *** *P* < .001.

## 3. Results

### 3.1. Identification of differentially expressed genes

**Figure 1.**
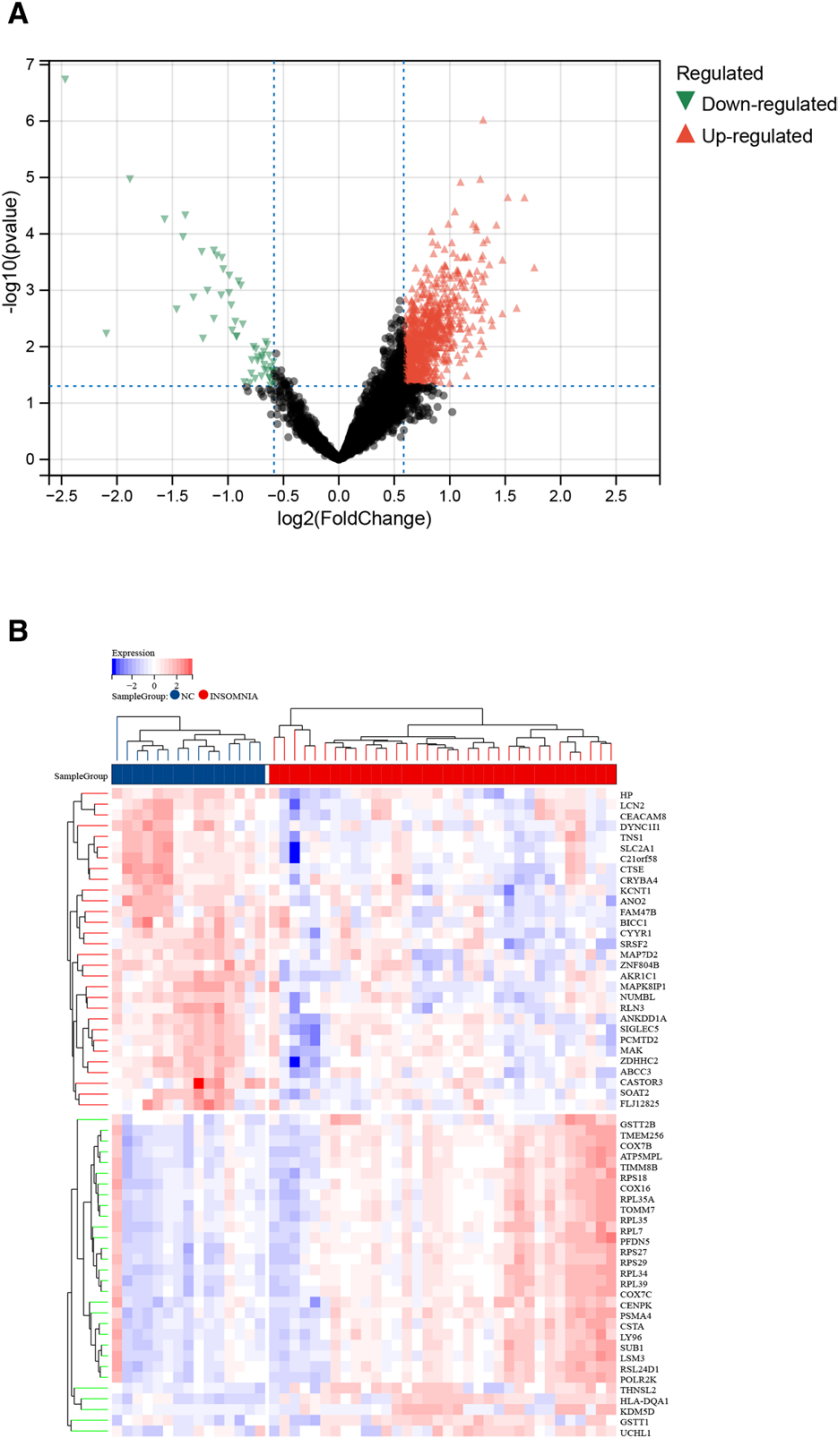
Heatmap and volcano plot for the DEGs identified from the integrated insomnia dataset. (A) Red and green plot triangles represent DEGs with upregulated and downregulated gene expression. (B) Each row displays DEGs and each column refers to one of the insomnia case or control group samples. Red and blue colors represent DEGs with up- and down-regulated gene expression. DEGs = differentially expressed genes.

We first selected GSE82114 from the GEO dataset for our study. To obtain an integrated dataset, we used the Limma rapid differential analysis method. The differential gene expression analysis identified a total of 903 differentially expressed genes (Fig. 1A), of which 849 genes were up-regulated (shown in red) and 54 genes were down-regulated (shown in green). The heat map depicted the expression values of the top 30 differentially expressed genes (deg) (Fig. 1B), as shown in Figure 1. The volcano and heatmap clearly showed that degs exhibited significantly different expression patterns in insomnia compared to controls. These findings highlight the profound alterations in gene expression associated with insomnia, providing a foundation for further investigation into the underlying biological mechanisms and potential therapeutic targets.

### 3.2. Weighted gene co-expression network analysis and key module identification

**Figure 2.**
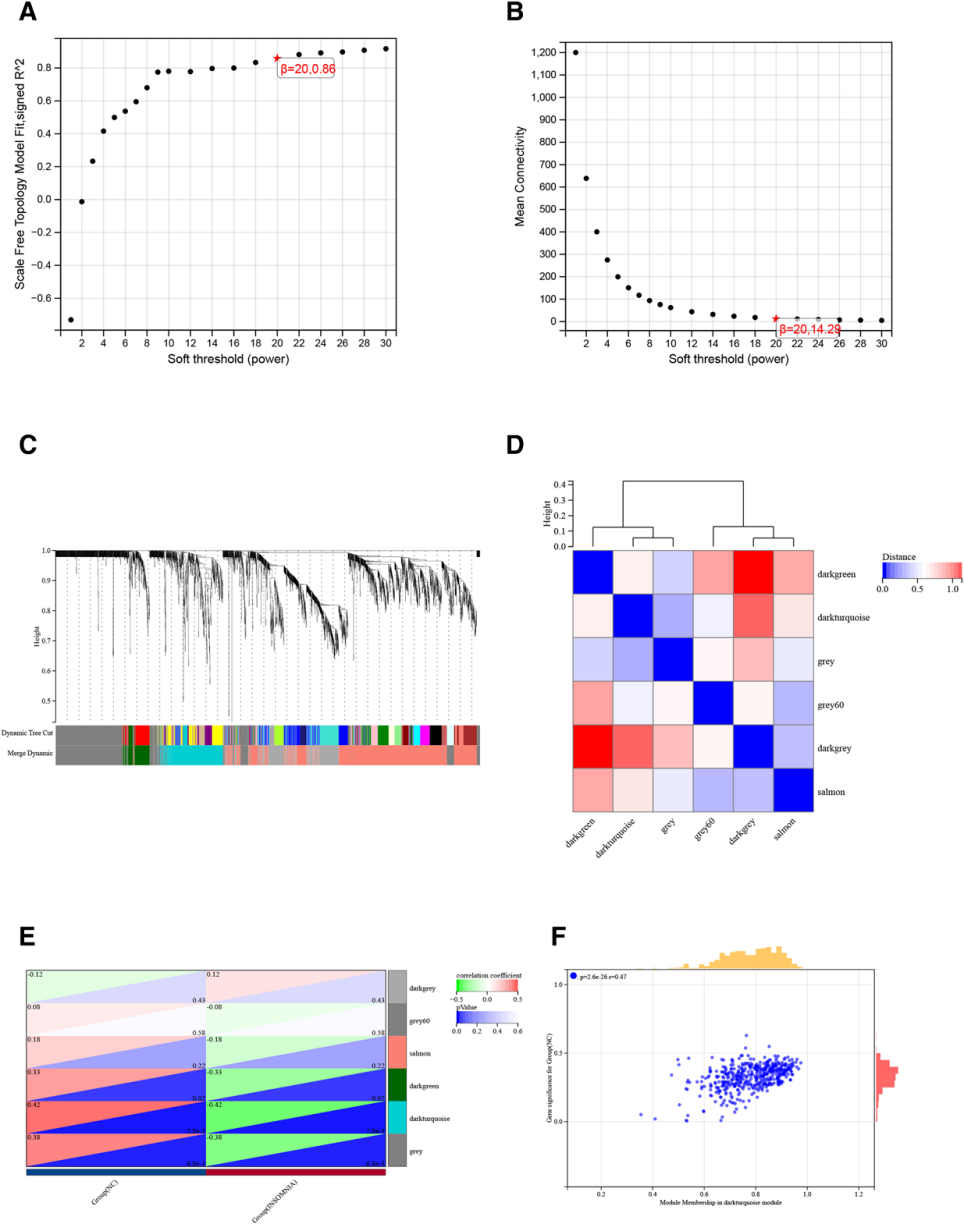
Weighted gene co-expression network (WGCNA) analysis. (A and B) Screening for soft thresholds based on scale independence and average connectivity. (C) Clustering dendrogram of the insomnia and control samples. (D) Heatmap of gene adjacency. (E) Heatmap of the association between modules and insomnia. The salmon module is shown to be correlated significantly with insomnia. Numbers at the top and bottom brackets represent the correlation coefficient and *P*-value. (F) Correlation plot between module membership and gene significance of genes included in the darkturquoise module.

To identify the most relevant modules for insomnia, the weighted gene co-expression network analysis (WGCNA) method was used in this study. In choosing the appropriate parameter values, we set a β value of 20 (scale-free *R*^2^ = 0.86) as a “soft” threshold based on scale independence and average connectivity metrics (Fig. 2A and B).

Figure 2C shows the gene clustering dendrogram for the normal and insomnia groups. Based on this clustering dendrogram, 6 gene co-expression modules were generated (Fig. 2D), each represented by a different color. To identify the module with the highest correlation with insomnia, we calculated the correlation between insomnia and each gene co-expression module (Fig. 2E). The results showed that the darkturquoise module had the highest correlation with insomnia (correlation coefficients of 0.42 *P* < .01, respectively). Therefore, we chose the darkturquoise module as the key module for subsequent analysis.

Next, we calculated the module member association between genes in the darkslateblue module and insomnia. The results showed a significant positive correlation between module membership and gene significance in this module (Fig. 2F, *R* = 0.47). We collected genes from this module for subsequent experiments. These analyses provide valuable insights into the gene networks associated with insomnia, highlighting potential targets for future research and therapeutic interventions.

### 3.3. Hub gene screening

**Figure 3.**
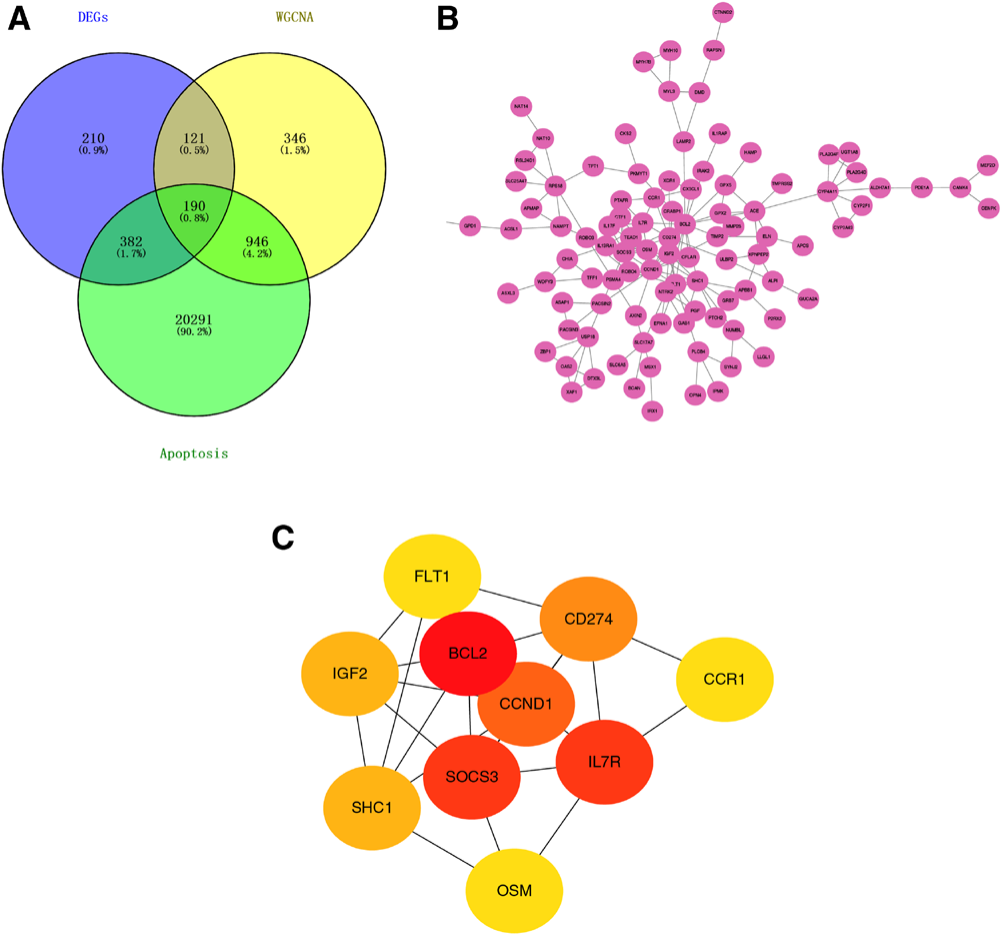
Hub gene screening and functional enrichment analysis. (A) Venn diagram showing the intersection between DEGs, Apoptosis gene database and key modular genes obtained by WGCNA analysis. (B) Apoptosis hub genes interaction in insomnia. (C) Ten hub genes screened by the cytoHubba plugin, of which 3 red genes are core hub genes. DEGs = differentially expressed genes, WGCNA = weighted gene co-expression network.

To construct a PPI network and screen the hub genes, we intersected the key module genes obtained from WGCNA analysis with DEGs as well as the apoptosis gene database to obtain 190 common genes (Fig. 3A), and then constructed a PPI network containing 190 common genes, which was input into Cytoscape for visualization (Fig. 3B). We then analyzed these 190 genes in cytoHubba software using the DNNC algorithm to identify the top 10 hub genes (Fig. 3C).

CytoHubba evaluates the importance of a node in the network by calculating its topological characteristics and centrality metrics. The shading color of a gene reflects its importance in the network. To identify these genetically important nodes more clearly, we chose to use red panels to highlight them. The red panels indicate nodes that may have important functions or key roles in the network. Based on these considerations, we chose the 3 genes with the highest Degree of red panels as hub genes, which are BCL2, SOCS3, and IL7R. These findings suggest that BCL2, SOCS3, and IL7R may play crucial roles in the molecular mechanisms underlying insomnia, and further investigation into these hub genes could provide insights for targeted therapeutic strategies.

### 3.4. Functional enrichment analysis of hub genes

**Figure 4.**
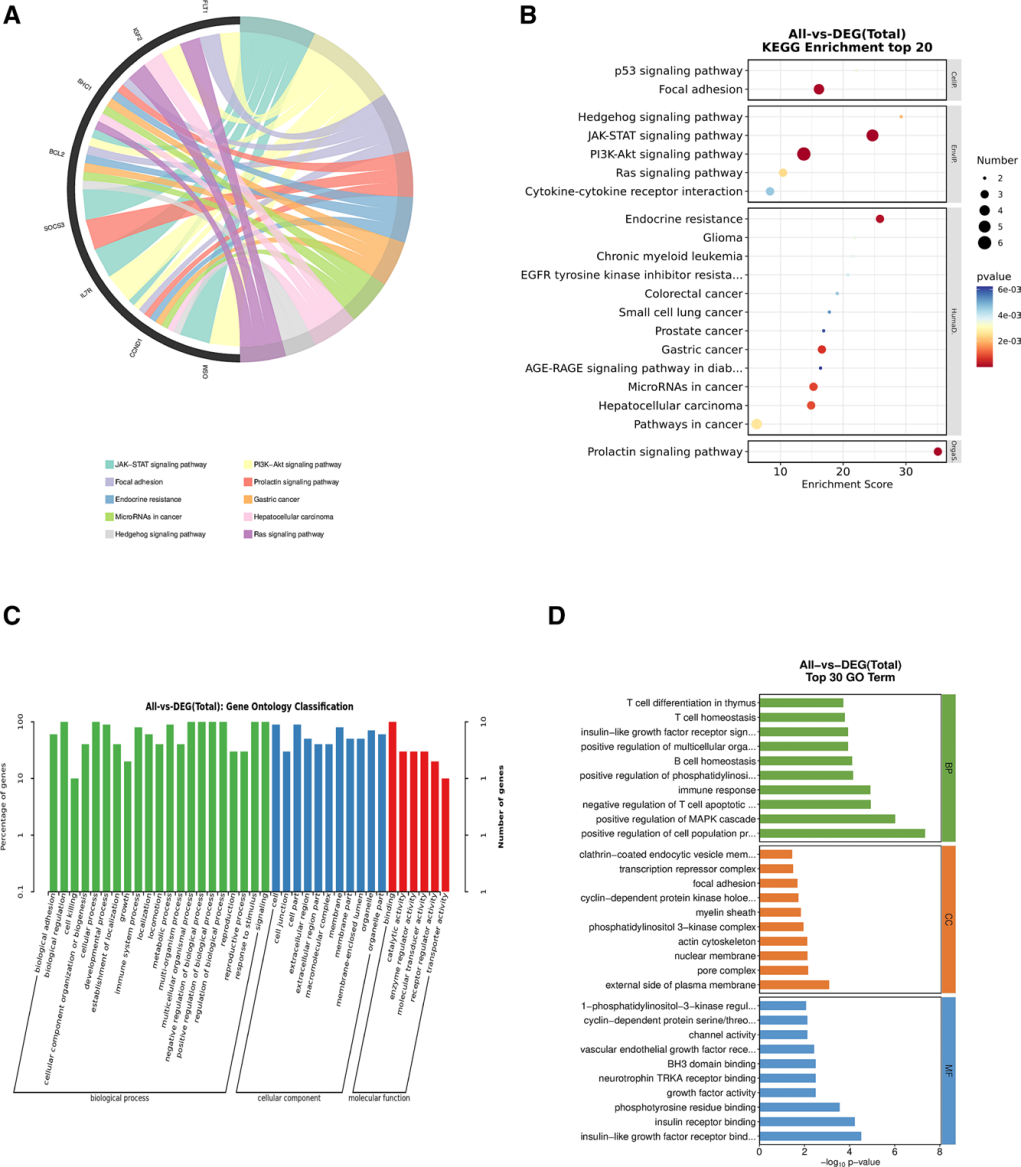
(A) Chord plot of KEGG enrichment analysis based on key gene set, (B) bar graph of KEGG enrichment analysis based on key gene set. (C) Signal categorization by GO enrichment analysis based on key gene sets, (D) top 20 signaling pathways by GO enrichment analysis based on key gene sets.

**Figure 5.**
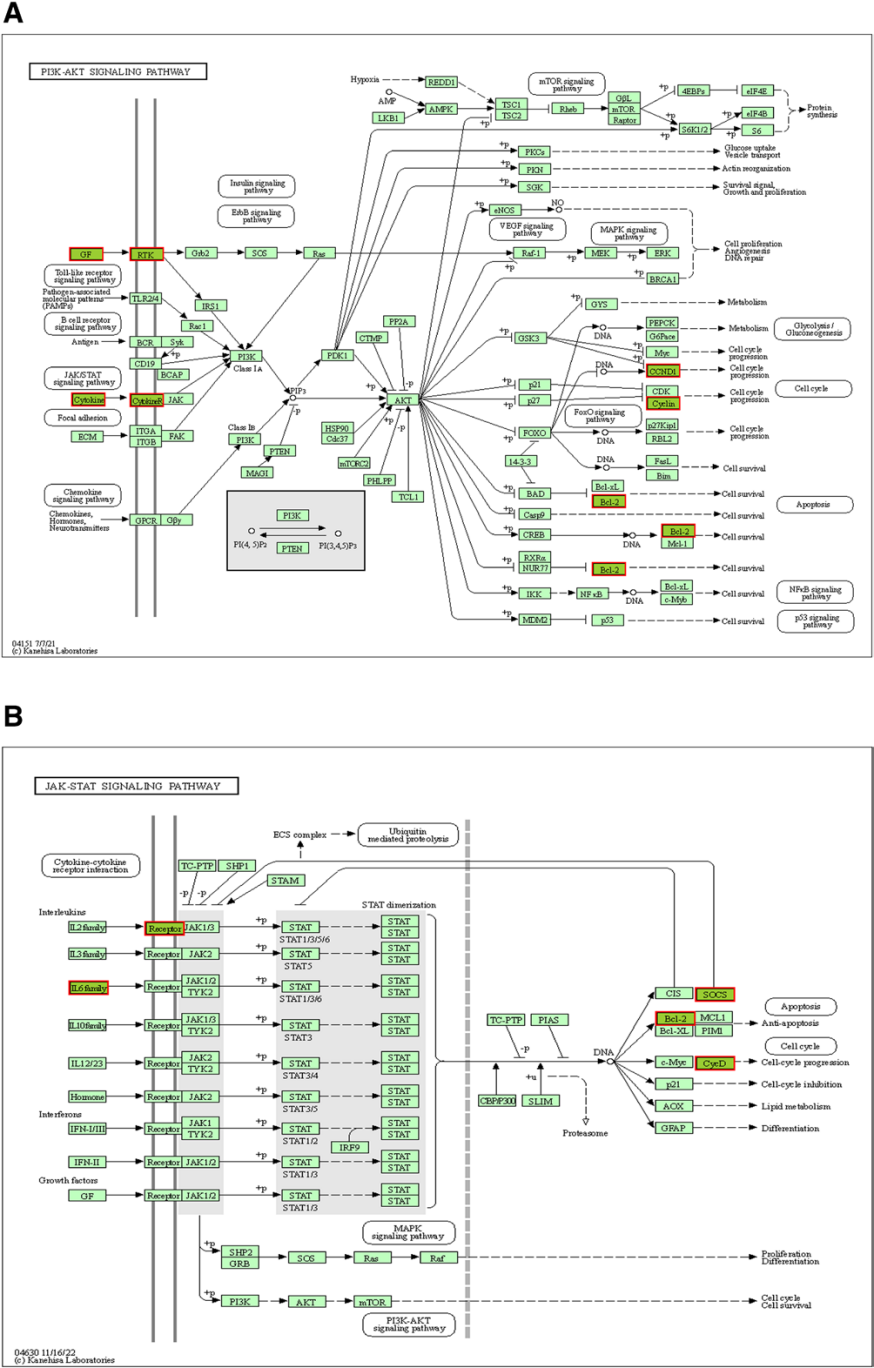
(A) Role of the key gene set in the signaling pathway in PI3K-AKT. (B) Role of the key gene set in the JAK-STAT signaling pathway.

To assess whether these 10 key genes reflect the pathogenesis of insomnia, we performed functional enrichment analysis. According to KEGG analysis, the 10 intersecting genes were mainly enriched in the “PI3K-Akt signaling pathway,” “JAK-STAT signaling pathway,” and “P53 signaling pathway,” which are shown as chordal bar graphs (Fig. 4A and B). GO analysis showed that it was mainly associated with biological processes such as “immune response,” “T cell differentiation in thymus,” “positive regulation of MAPK cascade” (Fig. 4C and D). The results showed that insomnia is closely related to the immune and apoptotic systems. The roles of these 10 key genes in the “PI3K-Akt signaling pathway” and “JAK-STAT signaling pathway” were demonstrated in Fig. 5.

### 3.5. GSEA analysis and expression characterization of the hub gene table

**Figure 6.**
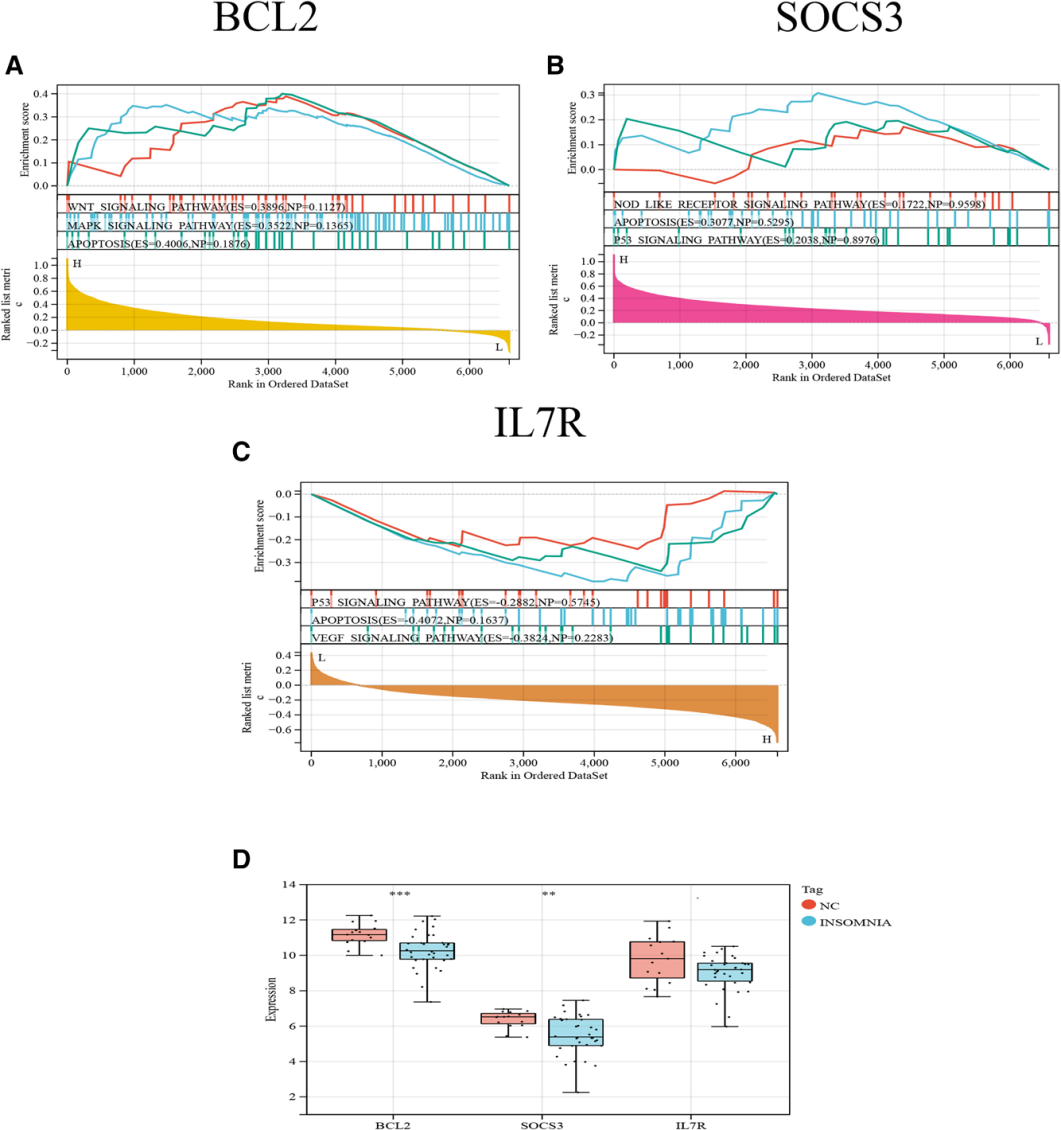
Apoptosis hub genes GSEA pathway analysis. (A) GSEA pathway analysis for BCL2. (B) GSEA pathway analysis for SOCS3. (C) GSEA pathway analysis for IL7R. (D) Box plot of the 3 hub genes. GSEA = Gene Set Enrichment Analysis.

GSEA analysis of BCL2, SOCS3, and IL7R using the dataset showed that BCL2 was enriched in “MAPK signaling pathway,” “Apoptosis signaling pathway” and “WNT signaling pathway,” SOCS3 was enriched in “P53 signaling pathway” and “Apoptosis signaling pathway,” while IL7R was enriched in the “P53 signaling pathway,” “Apoptosis signaling pathway” and “VEGF signaling pathway” (Fig. 6A–C). The results showed that BCL2, SOCS3, and IL7R were enriched in the apoptotic pathway. In conclusion, we used the data to show again that the development of insomnia is closely related to apoptosis. Further in the GEO82114 dataset, we verified the differential expression of these 3 hub genes in normal and insomnia samples. All 3 genes were lowly expressed in insomnia samples compared to controls (Fig. 6D). These findings reinforce the potential role of these hub genes in the pathophysiology of insomnia, suggesting that targeting the apoptotic pathways associated with these genes may offer new therapeutic avenues for managing insomnia.

### 3.6. WB and qRT-PCR experimental validation of hub genes

**Figure 7.**
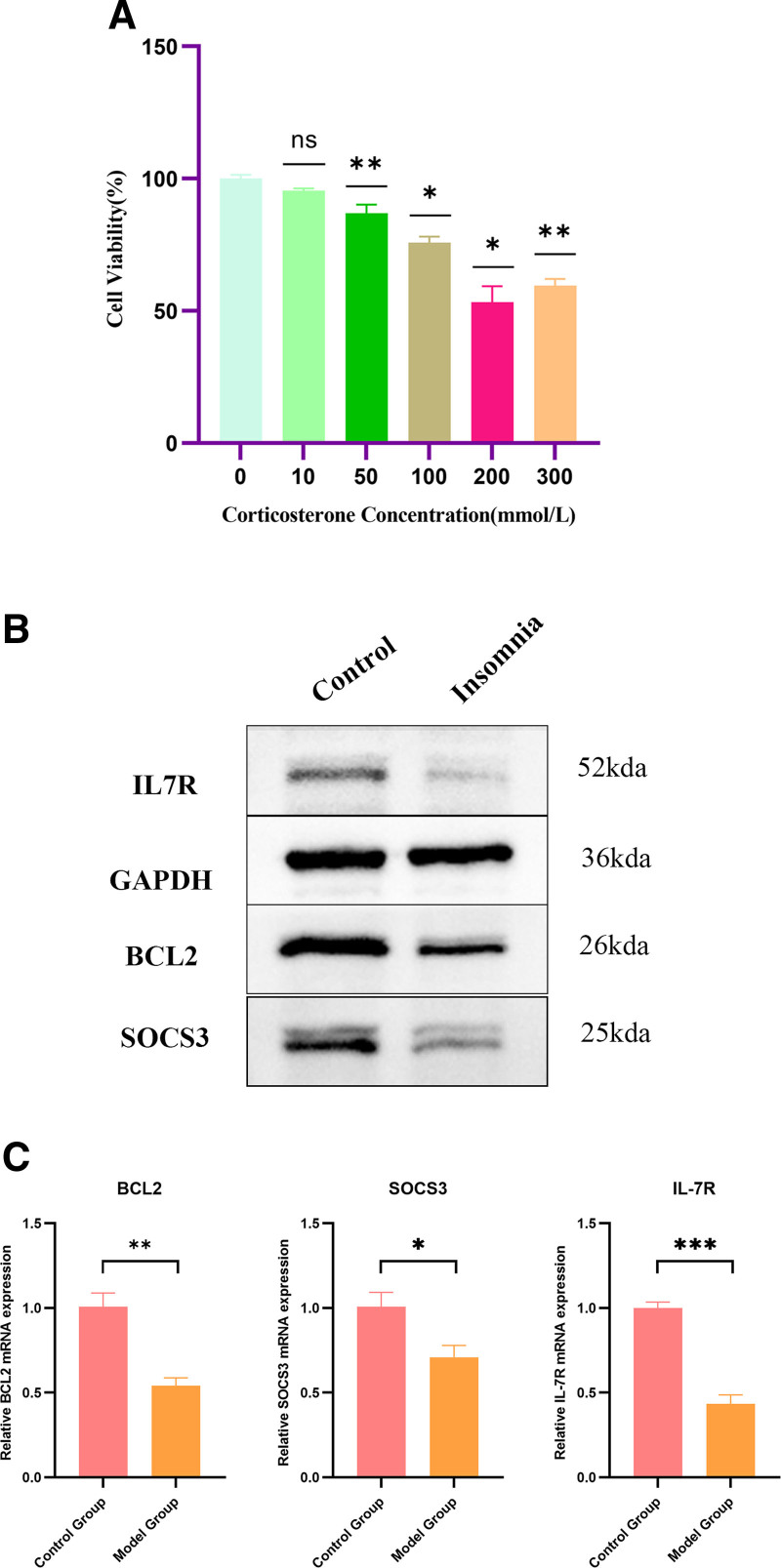
Identification and Western Blot, qRT-PCR validation of hub genes. (A) CCK8 detects the effect of different concentrations of corticosterone on the viability of hippocampal nerve cells. (B) Western Blot validation of the relative expression of the 3 hub genes between the control group of normal group and the insomnia model group (BCL2, SOCS3, IL7R). (C) qRT-PCR validation of the relative expression of the 3 hub genes between the control group of normal and the insomnia model group (BCL2, SOCS3, IL7R). *, *P* < .05, **, *P* < .01, ***, *P* < .001.

Through bioinformatic data analysis, we clarified that apoptosis signaling pathway plays an important role in the development of insomnia, and that the 3 hub genes, BCL2, SOCS3, and IL7R, are most likely involved in the development of insomnia. Based on this, we used normal hippocampal neuronal cells as a control group and intervened hippocampal neuronal cells using corticosterone as a model group, and a 200 mM concentration of corticosterone was determined as the optimal intervention concentration by CCK8 (Fig. 7A). We conducted WB experiments to detect the differences in protein expression levels of BCL2, SOCS3, and IL7R indicators in hippocampal neurons of normal control and insomnia model groups. The results showed that BCL2, SOCS3, and IL7R proteins were decreased in insomniac rats compared with the control group (Fig. 7B), which was consistent with the results of our previous analysis. Then we conducted qRT-PCR experiments for verification, and the results showed that BCL2, SOCS3, and IL7R genes also showed decreased expression in hippocampal neurons in the model group compared with the control group (Fig. 7C). In conclusion, we performed different experiments to validate the same conclusions. Importantly, the decrease in BCL2, SOCS3, and IL7R indexes indicated that the antiapoptotic capacity of hippocampal neurons in the insomnia model group was weakened, and thus it is highly likely that BCL2, SOCS3, and IL7R are important targets in the development of insomnia. Overall, these findings underscore the importance of apoptosis-related pathways in insomnia and suggest that targeting these pathways may provide new strategies for treatment.

## 4. Discussions

One of the most prevalent public health issues today is insomnia, which affects between 30% and 40% of adults.^[37]^ Short-term insomnia affects approximately 9.5% of the population, and 1 in 5 patients with short-term insomnia may develop chronic insomnia.^[38]^ Studies have found that the prevalence of insomnia is increasing year by year,^[39]^ insomniacs have a high risk of developing depression and anxiety within 1 year, and insomnia increases the risk of suicide in depressed patients.^[40,41]^ Currently, there are limited treatments for insomnia, and the underlying reason is that the pathophysiologic mechanisms of insomnia are not fully understood, so it is urgent to explore the mechanisms of insomnia. It has been shown that the occurrence of insomnia is directly related to neuronal apoptosis-induced inflammatory responses in the nervous system, and that sustained neuroinflammatory responses can induce neuronal apoptosis and cause central damage.^[42]^ Several neurobiological studies have shown a strong correlation between neuronal apoptosis and insomnia.^[43]^ Other studies have also shown that insomnia is associated with changes in the levels of lipid metabolites, which may adversely affect neuronal health and function. These changes promote apoptosis, further complicating the relationship between insomnia and its physiological effects.^[44–46]^ Bioinformatics studies can help us better understand complex diseases and have identified some genes that play a key role in the pathogenesis of insomnia.^[47]^ In this study, we mined 903 DEGs based on the GEO database and obtained 190 intersecting genes by intersecting with the key modules of WGCNA and the apoptosis gene database. We further screened the key genes and found that they were mainly enriched in “PI3K-Akt signaling pathway,” “JAK-STAT signaling pathway,” “P53 signaling pathway” and so on. GO analysis showed that it is mainly related to biological processes such as “immune response,” “T cell differentiation in thymus,” “positive regulation of MAPK cascade” and so on. In addition, we established a PPI network and utilized Cytoscape to obtain 3 hub genes, BCL2, SOCS3, and IL7R.

Apoptosis is initiated by internal or external stimuli and is mediated through 2 distinct pathways: the intrinsic pathway (mitochondria-mediated) and the extrinsic pathway (death receptor-mediated). The initiation of apoptosis^[48]^ is triggered by pro-apoptotic, BH3-only proteins, and BH3-only “activator” proteins include BIM (BCL-2 Interacting Mediator of Cell Death; encoded by BCL2L11), BID (BH3 Interacting Domain Death Agonist; encoded by BID), PUMA (p53 up-regulator of apoptosis; encoded by BBC3), among others,^[49]^ which bind to and activate the pro-apoptotic pore-forming proteins BAX (BCL-2-related X protein; encoded by BAX) or BAK (BCL-2 antagonist/killer; encoded by BAK1). Activation of BAX or BAK on the mitochondrial surface leads to a conformational change in these proteins, allowing them to oligomerize and form large pores in this membrane, leading to mitochondrial outer membrane permeabilization and subsequent release of apoptotic proteins from the membrane gap. In the cytoplasm, the released mitochondrial proteins can be directly or indirectly involved in cysteatase activation – for example, cytochrome c, which binds to the scaffolding protein APAF1 (Apoptosis Protease Activating Factor 1) to directly form apoptotic bodies, or SMAC, which neutralizes cysteatase inhibitory proteins to indirectly activate cysteatase to achieve the cell catabolizing breakdown effect.^[50]^

BCL2 is a key antiapoptotic protein that shakes up the apoptotic process described above, thus acting as an inhibitor of apoptosis. In the presence of insomnia, BCL2 expression may be modulated, thereby affecting neuronal survival, as observed by Liu Y et al who observed reduced BCL2 expression in the PCPA insomnia rat model.^[15]^ REM sleep deprivation may lead to changes in BCL2 expression, which may affect neuronal health and function and cause neuronal apoptosis.^[51]^ This suggests that BCL2 protein expression is decreased in insomnia disease, and the antiapoptotic effect it exerts is not sufficient to keep the process of apoptosis in balance, so much so that the insomnia disease shows the pathological manifestation of excessive apoptosis. Another study on ginsenoside Rg1 attenuating sleep deprivation damage showed that the up-regulation of BCL2 may help to attenuate the neuronal damage caused by insomnia and play a protective role.^[52]^ SOCS3 is a negative regulatory factor involved in cytokine signaling and immune responses. In states of insomnia, levels of inflammatory factors such as tumor necrosis factor-α are typically elevated, and SOCS3 helps alleviate inflammation by inhibiting these signaling pathways.^[53]^ Whereas other studies have shown that decreased SOCS3 expression induced apoptosis,^[54]^ SOCS3 overexpression reduced apoptosis.^[55]^ By regulating the expression of SOCS3, it may be possible to improve the inflammatory and apoptosis state associated with insomnia, leading to positive clinical outcomes. IL7R is an important component of the IL-7 signaling pathway and is involved in the development and survival of immune cells.^[56]^ Studies have shown that upregulation of IL7R expression stimulates the JAK1/STAT5 signaling pathway and enhances the production of the antiapoptotic protein BCL2.^[57]^ Insomnia may affect the expression of IL-7 and its receptor, thereby impacting immune system function.^[58]^ Changes in IL7R expression are closely related to mood disorders associated with insomnia, such as anxiety and depression.^[59]^ Enhancing the IL7R signaling pathway may help improve sleep quality while positively affecting the mental health conditions related to insomnia.^[58]^ In summary, the specific mechanisms of BCL2, SOCS3, and IL7R in insomnia interact through the regulation of apoptosis, inflammation, and immune responses, highlighting their significance in the pathophysiology of insomnia. These findings provide new targets for clinical treatment, potentially facilitating the development of therapeutic strategies for insomnia.

In this study, BCL2, SOCS3, and IL7R were all under-expressed in the hippocampal neurons of the insomnia model compared with the control group, suggesting a decreased antiapoptotic capacity. In conclusion, our study verified that apoptosis is closely related to the development of insomnia, and BCL2, SOCS3, and IL7R have the potential to be therapeutic targets for insomnia. Their specific mechanisms of action still need to be further explored. In future studies, I plan to explore novel forms of programmed cell death, such as pyroptosis, ferroptosis, and necroptosis. This will help ensure our research remains innovative and provide a more comprehensive perspective on the mechanisms related to insomnia.

It is worth noting that one of the key limitations of our study is the relatively small sample size, which may impact the generalizability of our findings. A larger sample size would provide more robust data and enhance the reliability of our conclusions. Additionally, our gene expression analysis is limited to specific databases. This restriction may introduce bias and limit the comprehensiveness of our findings. Future studies should incorporate a broader range of datasets to validate our results and explore gene expression patterns in more diverse populations.

## 5. Conclusions

Our study successfully identified key biomarkers and mechanisms associated with insomnia. We utilized a well-defined insomnia model, in which corticosterone was administered to hippocampal neurons to simulate conditions related to insomnia. Through the analysis of gene expression profiles, construction of protein–protein interaction networks, and experimental validation, we identified 3 pivotal hub genes: BCL2, SOCS3, and IL7R. These genes exhibited a trend of low expression in the hippocampal neurons of the insomnia model group, indicating a decrease in their antiapoptotic capacity. This finding suggests a significant relationship between apoptosis and the pathogenesis of insomnia.

Overall, our results indicate that BCL2, SOCS3, and IL7R may play important regulatory roles in the development of insomnia. This understanding deepens our insight into the pathophysiological processes of insomnia and provides potential targets and strategies for future clinical diagnosis and treatment.

## Author contributions

**Conceptualization:** Dechou Zhang.

**Data curation:** Xiao Wu.

**Software:** Nanxi Li, Bin Zhang.

**Visualization:** Lishan Huang, Hanxing Cheng.

**Writing – original draft:** Wenwen Zhu, Xingchun Yang.

**Writing – review & editing:** Sen Li, Houping Xu.
